# Factors Associated with Acute Malnutrition among Children Admitted to a Diarrhoea Treatment Facility in Bangladesh

**DOI:** 10.1155/2014/267806

**Published:** 2014-03-11

**Authors:** Connor Fuchs, Tania Sultana, Tahmeed Ahmed, M. Iqbal Hossain

**Affiliations:** ^1^University of Arkansas, Fayetteville, AR, USA; ^2^Centre for Nutrition and Food Security, and Nutrition Unit, Dhaka Hospital, icddr,b, Mohakhali, Dhaka 1212, Bangladesh

## Abstract

To assess the risk factors for acute malnutrition (weight-for-height *z*-score (WHZ) < −2), a case-control study was conducted during June–September 2012 in 449 children aged 6–59 months (178 with WHZ < −2 and 271 comparing children with WHZ ≥ −2 and no edema) admitted to the Dhaka Hospital of icddr,b in Bangladesh. The overall mean ± SD age was 12.0 ± 7.6 months, 38.5% (no difference between case and controls). The mean ± SD WHZ of cases and controls was −3.24 ± 1.01 versus −0.74 ± 0.95 (*P* < 0.001), respectively. Logistic regression analysis revealed that children with acute malnutrition were more likely than controls to be older (age > 1 year) (adjusted OR (AOR): 3.1, *P* = 0.004); have an undernourished mother (body mass index < 18.5), (AOR: 2.8, *P* = 0.017); have a father with no or a low-paying job (AOR: 5.8, *P* < 0.001); come from a family having a monthly income of <10,000 taka, (1 US$ = 80 taka) (AOR: 2.9, *P* = 0.008); and often have stopped predominant breastfeeding before 4 months of age (AOR: 2.7, *P* = 0.013). Improved understanding of these characteristics enables the design and targeting of preventive-intervention programs of childhood acute malnutrition.

## 1. Introduction

One of every five children aged less than 5 years in low-income, developing countries is malnourished. Globally, undernutrition is associated with more than one-third of all deaths in this age group [1]. Acute malnutrition defined by weight-for-height *z*-score (WHZ) < −2 (i.e., wasting) in young children continues to be a major health problem in low-income countries, particularly Bangladesh. Despite recent advances in prevention and management of childhood malnutrition in Bangladesh, 16% of children under 5 years of age are acutely malnourished (WHZ < −2) [2]. UNICEF describes malnutrition in Bangladesh as a “silent emergency” [3]. Childhood malnutrition places a heavy burden on many families in Bangladesh and other developing countries. It not only directly increases mortality but also imposes significant national health and development costs due to associated morbidities, including impaired cognitive ability and indirect deaths.

It is well documented that poverty and malnutrition, regardless of location, are highly intertwined. Although risk factors for malnutrition have been identified, individual factors potentially change in specific areas over time and a current characterisation of risk factors provides the basis for preventative intervention programmes.

## 2. Methods

The study was conducted in the Dhaka Hospital of the International Centre for Diarrhoeal Disease Research (icddr,b) situated in Dhaka, Bangladesh, a metropolitan area (1,500 sq km) with a total population of ~15 million. Each year, the Dhaka Hospital provides care and treatment for over 120,000 patients with diarrhoea, with or without other associated health problems. The hospital also conducts research on enteric and other common infectious diseases as well as undernutrition and provides training on case management of diarrheal diseases, management of malnutrition, and research methodology. Under-five children constitute about 60% of the total patient population and most (~60%) are from poor socioeconomic communities in urban and periurban areas of Dhaka.

A nonmatched case-control study design was used to assess and identify potential risk factors associated with acute malnutrition/wasting among 6–59-month-old children. Children in this age group without any congenital anomaly or other chronic conditions causally associated with malnutrition (e.g., heart disease), who were admitted to the Dhaka Hospital of icddr,b from June to September 2012, were enrolled. Using the contemporary growth standards of the World Health Organization (2006) and ANTHRO software [4], weight-for-height *z*-score (WHZ), weight-for-age *z*-score (WAZ), height-for-age *z*-score (HAZ), and body mass index-for-age *z*-score (BMI-AZ) of all studied children were calculated. Children with moderate or severe wasting (WHZ < −2) but without bilateral pedal edema were considered cases. Children with a WHZ ≥ −2 and without bilateral pedal edema were enrolled as control children. Verbal consent from the attending guardian, usually the mother of the child, was obtained.

### 2.1. Data Collection

One of the investigators and/or a research assistant interviewed the mother/caregiver using a pretested, structured questionnaire. Data recorded from the interviews included age and sex of child, birth order, number of total and under-five siblings, feeding and immunisation history, type of house hold latrine, marital status of mother, monthly family income, and parental age, education, and occupation. Children's nude weight using a frequently standardised digital scale with 10 g precision (Seca, model 345, Hamburg, Germany) and recumbent length to the nearest mm using a calibrated, locally constructed length board were obtained. Mother's (if present) weight and height were measured using standard procedures [5]. The investigator(s) supervised the interview process and anthropometry and reviewed the data forms daily.

### 2.2. Data Analyses

Data were entered using SPSS software for Windows (version 11.5) (SPSS Inc., Chicago, IL, USA). For normally distributed continuous variables, means were compared using unpaired *t*-tests. For continuous variables not normally distributed, the Mann-Whitney *U* test was performed. Differences in proportions were compared by the chi-square test or Fisher's exact test if the expected number in any cell was ≤5. A probability of less than 0.05 was considered statistically significant. The strength of association of selected associated/risk factors for acute malnutrition was determined by estimating odds ratios (ORs) and their 95% confidence intervals (CIs). All independent variables, for example, birth order, number of siblings, socioeconomic status, parental characteristics, child feeding, and immunisation history, were analysed initially in univariate models and the attributes that were significantly associated with wasting (dependent variable) and biologically plausible were included in logistic regression models.

## 3. Results

A total of 449 children were enrolled, of whom 178 were cases (wasted children) and 271 were controls. Their overall mean ± SD age was 12.0 ± 7.6 months (cases were on an average 2 months older), and 38.5% were female without any group difference between case and control children. The mean ± SD WHZ and BMIAZ of case and control children were −3.24 ± 1.01 versus −0.74 ± 0.95 and −3.21 ± 1.04 versus −0.77 ± 0.96, respectively (*P* < 0.001). As dictated by the study design, the cases had significantly lower anthropometric values than the control children (Table 1). Cases had a shorter period than controls of exclusive or predominant breastfeeding. Maternal nutritional status, family income, and the fathers' educational levels were worse in the malnourished children (cases). Cases had worse immunization status (Bacillus Calmette-Guérin (BCG) and measles vaccine), breastfeeding duration, and parental and other socioeconomic characteristics than control children (Table 2). Logistic regression analysis revealed that children with acute malnutrition were more likely than control to be older (age > 1 year) (adjusted OR (AOR): 3.1, *P* = 0.004), have an undernourished mother (body mass index (BMI) < 18.5), (AOR: 2.8, *P* = 0.017), have a father with no or a low-paying job (AOR: 5.8, *P* < 0.001), come from a family having a monthly income of less than 10,000 taka, (1 US$ = 80 taka) (AOR: 2.9, *P* = 0.008), and have shorter period of predominant breastfeeding (AOR: 2.7, *P* = 0.013) (Table 3).

## 4. Discussion

The aim of this study was to identify risk factors associated with acute malnutrition (WHZ < −2, which includes both moderate and severe wasting) in our population of 6–59-month-old children. Our study shows that the major associated/risk factors for acute malnutrition among these children were older age of the child, undernourished mother, jobless father or father with a low-paying job, low total family income, and poorer breastfeeding practices. Some of these factors may operate in synergy to increase the risk of acute malnutrition.

Older age as a risk/associated factor for acute malnutrition of children in our study might reflect a selection bias. However, Jeyaseelan and Lakshman from Tamil Nadu, India [6], and Kikafunda et al. from Uganda [7] also observed older age of a child to be significantly associated with malnutrition. Proper nutrition and child care are essential to begin in early childhood to prevent malnutrition and ensure maximum potential for normal psychomotor development as children grow older.

Similar to other studies from Bangladesh [8–11] and Africa [12], the current study observed maternal malnutrition (BMI less than 18.5) as an independent risk factor for children's wasting/acute malnutrition. Undernourished mothers often deliver low-birth-weight (LBW) infants [13], which is an attributable risk factor for increased childhood malnutrition, morbidity, and mortality. The last nationwide LBW survey in Bangladesh showed a high prevalence of 36%. The capacity to adequately breastfeed may also be compromised [14–16] in maternal malnutrition and such mothers might have less energy to appropriately nurture their children.

The fathers of most (84%) of the wasted children in our study were rickshaw pullers or day laborers. Likewise, a study from South India [17] also found that children of fathers who were day laborers were ~3 times more likely to be severely underweight. These occupations are among the lowest paid employment categories in Bangladesh. Moreover, they often result in erratic or insecure incomes, at times yielding little or no earnings on a particular day. Income insecurity leads to food insecurity forcing family members to consume poor food quality and/or amount. Low family income as a possible risk factor of acute malnutrition, as found in this study, was also reported by Ahmed et al. [8] in wasted children under 2 years old, by Nahar et al. [18] in severely underweight under-5 children in Bangladesh, and by Jeyaseelan and Lakshman [6] in undernourished children in Tamil Nadu, India.

Our finding of improper/inadequate breastfeeding as an associated factor with acute malnutrition is in accordance with the findings of several other studies [10, 19, 20] from Bangladesh and elsewhere, in which early supplementation with infant formula or cow's milk, early introduction of semisolid complementary foods, and inadequate breastfeeding were important risk factors for malnutrition in children. During the critical period of early infancy, proper breastfeeding and complementary feeding practices play critical roles. The hygienic and nutritional risks associated with bottle-feeding and artificial milk are well known [21–23], and previous studies also found that breastfeeding had a significant and substantial impact on overall survival of undernourished children [24, 25]. It is also possible that the association with a shorter duration of predominant breastfeeding could be an example of reverse causality, whereby children who were ill and undernourished stopped breastfeeding or were provided with other foods.

One of the possible limitations of the present study is that the same personnel who obtained the anthropometric measurements of the children and mothers also conducted the interviews, so interviewer biases could be there. However, most of the variables identified as risk/associated factors for acute malnutrition in our study were objective in type. The other limitation was the cross-sectional nature of the present study, which did not allow us to state the identified associated factors as definite causally related risk factors.

The children suffering from acute malnutrition often need supplementary food. In this regard it is worthwhile to mention that icddr,b has developed ready-to-use foods using locally available food ingredients. These foods can be used to prevent and to treat moderate wasting in children living in food-insecure communities. Moreover, the associated factors identified for acute malnutrition in this study can be incorporated into the design and targeting of preventive interventions. Factors such as breastfeeding practices are potentially modifiable. Interventions that motivate behaviours more consistent with recommended infant and young child feeding practices would be expected to have a positive impact. Certain factors and possible causes of acute malnutrition are complex and involve societal and broad-based preventive programs.

## Figures and Tables

**Table 1:** Characteristics (continuous variables) of the cases (wasted) and controls (nonwasted) children.

Variable	Case *N* = 178	Control *N* = 271	*P* value
Child's age (months)	13.4 ± 4.0	11.2 ± 6.6	0.004
Weight-for-length *z*-score^a^	−3.24 ± 1.01	−0.74 ± 0.95	<0.001
Weight-for-age *z*-score^a^	−3.46 ± 1.34	−1.08 ± 1.19	<0.001
Length-for-age *z*-score^a^	−2.16 ± 1.84	−0.88 ± 1.45	<0.001
BMI-for-age *z*-score^a^	−3.21 ± 1.04	−0.77 ± 0.96	<0.001
Total number of children in the family	1.8 ± 1.0	1.7 ± 0.9	0.270
Exclusive/predominant breastfeeding (month)	4.0 ± 2.6	4.4 ± 2.4	0.042
Birth order	1.8 ± 1.0	1.7 ± 0.8	0.365
Mother's age (years)	23.7 ± 5.2	23.9 ± 4.7	0.737
Mother's weight (kg)	45.0 ± 7.8	50.4 ± 10.2	<0.001
Mother's height (meter)	1.49 ± 0.05	1.50 ± 0.06	<0.001
Mother's body mass index (kg/M^2^)	20.4 ± 3.4	22.3 ± 4.0	<0.001
Mother's education (years)	5.0 ± 4.2	7.5 ± 3.9	<0.001
Father's age (years)	30.8 ± 6.8	31.5 ± 5.7	0.249
Father's education (years)	5.8 ± 4.6	8.3 ± 4.3	<0.001
Total family income per month (taka^b^)	10128 ± 6647	16095 ± 16489	<0.001

All data are expressed as mean ± SD. ^a^In relation to the WHO 2006 standard [4]; ^b^taka (Bangladeshi currency: 1 UD$ = 80 taka, average rate during the study period).

**Table 2:** Characteristics (attributes) of the cases (wasted) and controls (nonwasted) children.

Variable	Case *N* = 178	Control *N* = 271	*P* value
Child's age > 1 year: *n* (%)	67 (37.6)	62 (22.9)	0.001
Girls: *n* (%)	74 (41.6)	99 (36.5)	0.283
Did not receive BCG: *n* (%)	5 (2.8)	1 (0.4)	0.038
Did not receive pentavalent/polio vaccine (or received less than age appropriate doses): *n* (%)	16 (9.0)	23 (8.5)	0.984
Did not receive measles vaccine (among >9 months old; *n* = 109 cases and 142 controls): *n* (%)	31 (28.4)	26 (18.3)	0.041
Predominant breastfeeding stopped before 4 months: *n* (%)	76 (42.7)	85 (31.4)	0.010
Teenaged mother (<20 years): *n* (%)	23 (13.4)	21 (7.9)	0.045
Shorter mother (height < 1.5 meters): *n* (%)	86 (54.4)	110 (44.5)	0.033
Undernourished mother (BMI < 18.5): *n* (%)	52 (32.5)	37 (14.9)	<0.001
Illiterate or less educated (<5 years' schooling) mother: *n* (%)	101 (56.7)	88 (32.5)	<0.001
Mother working outside of the home: *n* (%)	15 (8.4)	12 (4.4)	0.063
Divorced/widowed mother: *n* (%)	16 (9.0)	3 (1.1)	<0.001
Younger father (age < 25 years): *n* (%)	44 (25.4)	37 (14.3)	0.003
Illiterate or less educated (<5 years' schooling) father: *n* (%)	92 (52.6)	72 (27.0)	<0.001
Father with low-paid job: *n* (%) (e.g., rickshaw puller or day labourer): *n* (%)	149 (83.7)	139 (51.3)	<0.001
Monthly income < 10000 taka^a^: *n* (%)	121 (70.3)	136 (51.5)	<0.001
Using unsanitary latrine: *n* (%)	10 (5.6)	3 (1.1)	0.006
Child worn any thread or amulet: *n* (%)	94 (53.1)	157 (57.9)	0.331
Child worn kajal^b^ at the side of fore head: *n* (%)	139 (78.1)	219 (80.8)	0.549

All data are expressed as number (%). ^a^Taka (Bangladeshi currency: 1 UD$ = 80 taka, average rate during the study period); ^b^a black mark/line used over eyelash by females and side of forehead in some children in Indo-Pak subcontinent.

**Table 3:** Factors associated with acute malnutrition (wasting): results of logistic regression model.

Attribute	Adjusted odds ratio	95% CI of adjusted OR		*P* value
		Lower	Upper	
Child's age > 1 year	3.144	1.431	6.904	0.004
Did not receive measles vaccine (among >9 months old; *n* = 109 cases and 142 controls)	2.492	0.973	6.378	0.057
Predominant breastfeeding stopped before 4 months	2.669	1.229	5.796	0.013
Teenaged mother (<20 years)	1.758	0.451	6.847	0.416
Shorter mother (height < 1.5 meters)	1.399	0.686	2.851	0.355
Undernourished mother (BMI < 18.5)	2.803	1.203	6.532	0.017
Illiterate or less educated (<5 years' schooling) mother	1.676	0.754	3.728	0.205
Younger father (age < 25 years)	1.614	0.682	3.815	0.276
Illiterate or less educated (<5 years' schooling) father	1.186	0.525	2.682	0.681
Father with low-paid job	5.778	2.537	13.157	<0.001
Monthly income < 10000 taka ^a^	2.871	1.310	6.291	0.008
Using unsanitary latrine	1.505	0.078	29.173	0.787
Constant	0.016	—	—	0.001

^a^Taka (Bangladeshi currency: 1 UD$ = 80 taka, average rate during the study period).
